# Insight into the microbial diversity and community in the sacrificial pits of Sanxingdui site (Sichuan, China)

**DOI:** 10.3389/fmicb.2024.1489025

**Published:** 2024-12-10

**Authors:** Ruru Chen, Zhenbin Xie, Qing Xiao, Chong Wang, Rui Wen

**Affiliations:** ^1^School of Cultural Heritage, Northwest University, Xi’an, China; ^2^Sichuan Institute of Cultural Relics and Archaeology, Chengdu, China

**Keywords:** Sanxingdui site, high-throughput sequencing, microbiome, biodeterioration, burial activity

## Abstract

**Introduction:**

The Sanxingdui site (Sichuan, China) is the typical representative of the ancient Shu culture, which lasts from the late Neolithic to early Western Zhou. The sacrificial pits are located in the core region of Sanxingdui site, and numerous artifacts are unearthed including ivory, seashells, bronzes, pottery, jade, stone, gold, bone, and horn products. The function of the pits and buried artifacts has always been the focus, but the microbiome around artifacts attracts less attention. Recently, the microbiome in buried ivory soil has just been identified; however, the microbiome around other artifacts has never been studied. In term of the unique perspective for interpretation the archaeological issues, the study was carried out for: (1) the microbial diversity and community of soil in the lower layer of artifacts in sacrificial pits, (2) the potential biodeterioration behavior of organic and inorganic relics, and (3) the impact of sacrificial and burial activities in different sacrificial pits on microbiome.

**Methods:**

There were 45 soil samples around different artifacts in three sacrificial pits and 12 raw soil samples inside or outside the sacrificial pit sampling from Sanxingdui site. The microbial genomes were then identified and analyzed using the next-generation high-throughput sequencing.

**Results:**

The represented bacterial phyla were Proteobacteria, Actinobacteriota, GAL15, Chloroflexi, Acidobacteriota, Methylomirabilota, Thermoplasmatota, Crenarchaeota, Gemmatimonadota, and Firmicutes, and the represented fungal phyla were Ascomycota, Mortierellomycota, and Basidiomycota. Further microbial functional analysis found that the bacterial genera *Sphingopyxis*, *Limnobacter*, and *Streptomyces* and the fungal genera *Cladosporium*, *Acremonium*, and *Mortierella* were concerned with the degradation of organic matter, while the genera *Pseudomonas, Arthrobacter*, *Variovorax*, *Aspergillus*, and *Penicillium* might be related to the biocorrosion of bronzes. In addition, the microbial composition and principal co-ordinate analysis (PCoA) demonstrated the significant differences in microbial composition and structure between the raw soil samples and the soil samples around the artifacts and also between the soil samples in different sacrificial pits.

**Discussion:**

It is important to understand the biodeterioration of the buried artifacts and the sacrificial activities in Sanxingdui site according to the results of microbial diversity and community. The combination of microbiology and archaeology will shed light on the archaeological issues related to the ancient human activities and behaviors.

## 1 Introduction

Microbes are ubiquitous and are isolated from various archaeological relics and cultural heritage, including bones (Kazarina et al., 2019), dental calculi (Gancz et al., 2023), mural (He et al., 2021), wooden relics (Liu et al., 2018; Cao et al., 2024), historical textiles (Brzozowska et al., 2018), stone relics (Liu et al., 2020), metallic antiquities (Junco et al., 1992), and pottery (Ren et al., 2023). The combination of archaeology and microbiology promises to provide an innovative perspective for the analysis and interpretation of archaeological issues. On the one hand, microbial diseases of cultural heritage have always been a research focus in the field of archaeological heritage conservation (Li et al., 2017; He et al., 2021; Zhang et al., 2023). The degradation and deterioration caused by microbial activity are non-negligible and irreversible during the long-term burial or open environments, resulting in the destruction of economic, artistic, historical, and cultural value. The organic matter is attacked and broken down by microbial metabolic pathways and enzyme action, which is converted into simple organic acids, amino acids, glucose and carbonates, etc., accelerating the energy flow and nutrient cycle in the ecosystem (Yang et al., 2024). The most common mechanism of biodeterioration on inorganic and metallic is concerned with the formation of bacterial and fungi biofilms, which are composed of cells, spores, fat globules, and dirt matter, and gradually hardened into the sticky layers through the action of cellular excretions (Swaroop et al., 2016). Numerous microbes are inhabited in the biofilms, causing the physical and chemical damage of metal, stone, or pottery relics via the release of various bioactive natural products.

On the other hand, microbial community in the soil of archaeological sites can serve as certain indicators of ancient human activities. Some studies indicate that human consumption and behavior habits leave traces on microbiome in archaeological evidence (Margesin et al., 2016; Siles et al., 2018). Therefore, the differences of microbial composition and structure at different ancient human-impacted soil regions can be regarded as the unique record of environmental changes and anthropogenic activities during their formation, providing necessary information to archaeological research over long time scales. Recently, the microbial community inhabiting in soil from three archaeological human-impacted layers at Monte Iato settlement (Sicily, Italy) has been investigated and proved the distinction on microbial C-source utilization patterns and structures reflecting different past human activities (Siles et al., 2018). But the microbiome of archaeological soil has received less attention than other remains. It is extremely necessary to identify the soil microbial community of buried environments not only for assessing microbial biocorrosion of burial artifacts but also for speculating on human activities and burial behaviors at the time.

**Figure 1:**
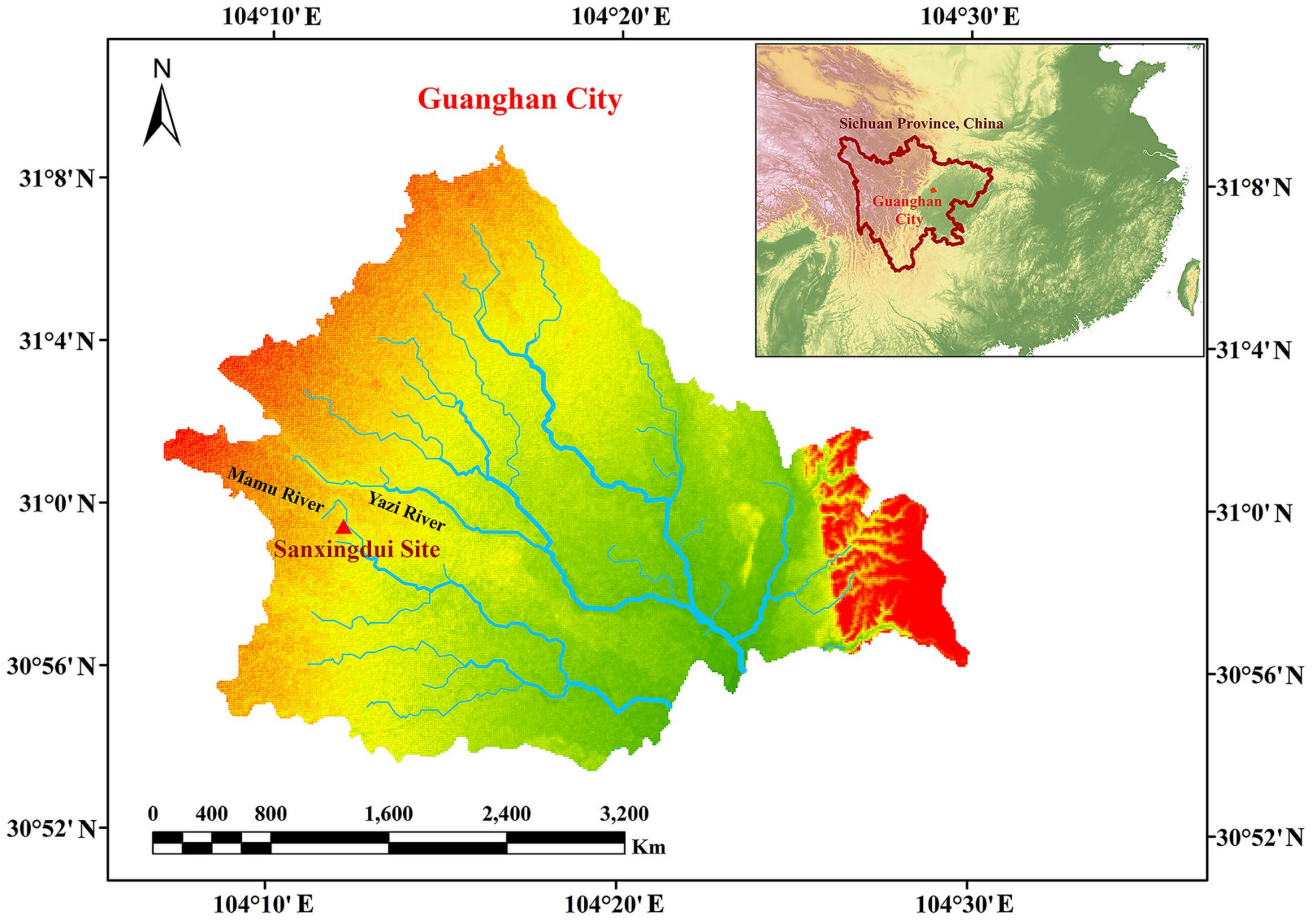
Map of Sanxingdui site.

**Figure 2:**
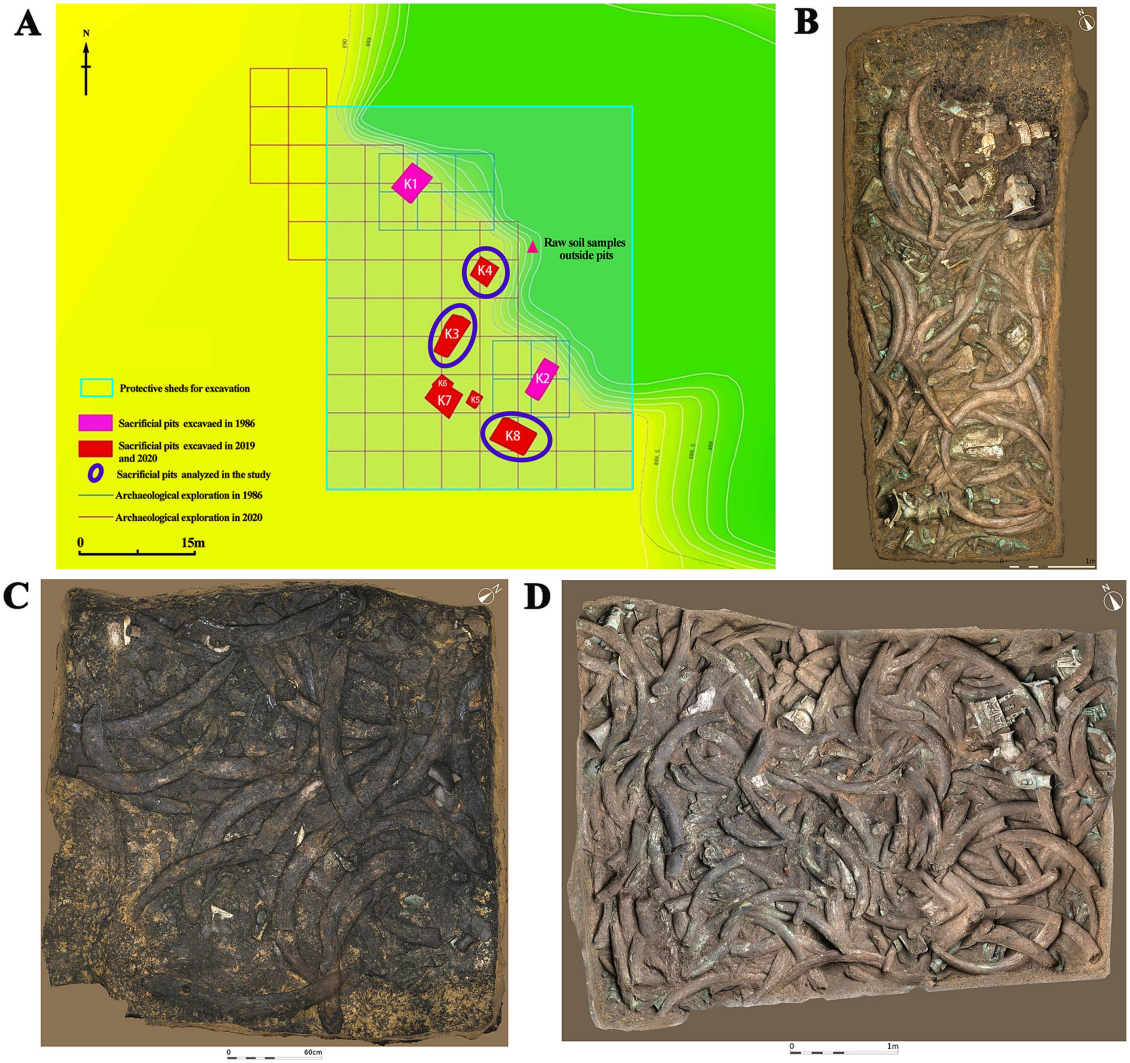
Distribution of sacrificial pits and images of artifacts in analyzed sacrificial pits. **(A)** Distribution of sacrificial pits. **(B)** Sacrificial pit K3. **(C)** Sacrificial pit K4. **(D)** Sacrificial pit K8.

The Sanxingdui site (Figure 1) is located in Sanxing Village, Sanxingdui Town, Guanghan City, Sichuan Province, China, on the south bank of the Yazi River and in the western suburbs of Guanghan City. The site is regarded as an outstanding representative of the ancient Shu culture for the late Neolithic to early Western Zhou, selecting as one of the “Top 100 Archaeological Discoveries of the Century” in China. The sacrificial area is situated on the south bank of the Mamu River, covering an area of approximately 13,000 cm^2^. In 1986, two sacrificial pits, named No. 1 pit (K1) and No. 2 pit (K2), were discovered in the first archaeological excavation. Until 2019, six newly discovered sacrificial pits (numbered K3 to K8) were found and excavated in 2020. The brief excavation reports of the sacrificial area and sacrificial pit K4 have been published, respectively, confirming that the ^14^C dating of K4 is 3,148–2,966 cal. BP. The artifacts unearthed from the sacrificial pits include ivory, seashells, bronzes, pottery, jade, stone, gold, bone, and horn products. The distribution and artifacts buried in K3, K4, and K8 are shown in Figure 2. The deposits of K3 and K4 are stratigraphically divided into rammed earth, ash layer, and artifact layer, while deposit of K8 is more complex and divided into rammed earth, ash layer, separation layer, and artifact layer. In the artifact layer of K3, K4, and K8, the upper is the ivory, and the lower is the other artifacts involving bronzes, gold, jade, pottery, etc. The microbial community of the ivory soil has been detected in previous study (Sun et al., 2024). But the microbial composition and structure in the lower soil of artifact layer have received little attention.

The aim of the study was to identify the microbial community in the lower soil of artifact layer via high-throughput sequencing and then to discuss the effect of sacrificial ceremony, sacrifice selection, burial activities, and other activities at that time on microbial composition and structure, simultaneously, to further evaluate the possible biodeterioration of the buried artifacts, providing a basis for the long-term preservation.

## 2 Materials and methods

### 2.1 Archaeological site and sample collection

Archaeological excavations of the six new sacrificial pits K3 to K8 were conducted in October 2020. The sacrificial pits K3, K4, and K8 were selected as the subject of this study. The shape, size, and some physical–chemical properties of the three sacrificial pits were introduced in the previous reports (Sun et al., 2024). A brief introduction to soil moisture content and pH value of buried environment was as follows. The soil moisture content in K3 was 22.64%, and the pH was 7.16. The soil moisture content of K4 was 22.08%, and the pH was 7.18. The soil moisture content of K8 was 20.20%, and the pH was 7.19. During the excavations, five soil samples (numbered “1–5,” test sample) were collected from the lower of artifact layer in K3, K4, and K8, respectively, for a total of 15 samples. In addition, the raw soil samples (numbered “0,” control group A) at the bottom of each pits in K3, K4, and K8 and one raw sample (numbered “S_0,” control group B) outside the sacrificial pits were also analyzed as control samples to comparative analysis. Soil samples were gathered using sterile scalpel and collected into Corning centrifuge tubes with −30°C storage temperature. As shown in Tables 1, 19 samples were categorized according to the excavated sacrificial pits and the type of artifacts near its location. All samples were sequenced three replicates to minimize errors and finally a total of 57 samples.

**Table 1:** Soil samples of sacrificial pits in Sanxingdui site.

Group	Name	Type	Sampling location	Brief description
K3_0	K3_0	Control group A	Raw soil at the bottom of southeast of K3	——
K3	K3_1	Test group	Soil near a bronze figure and a jade object	——
	K3_2	Test group	Soil from the inner abdomen of a bronze *dakouzun*	Near bronze
	K3_3	Test group	Soil at the top of a bronze figure	Near bronze
	K3_4	Test group	Soil near a bronze eye-shaped object	Near bronze
	K3_5	Test group	Soil at the bottom of a bronze figure with a *Zun*-vessel on its head	Near bronze
K4_0	K4_0	Control group A	Raw soil at the bottom of southwest of K4	——
K4	K4_1	Test group	Soil near a pottery sherd	Near pottery
	K4_2	Test group	Soil near a stone ware	——
	K4_3	Test group	Soil near a bronze ware	Near bronze
	K4_4	Test group	Soil near a bronze ware	Near bronze
	K4_5	Test group	Soil near a pottery sherd	Near pottery
K8_0	K8_0	Control group A	Raw soil at the bottom of northeast of K8	——
K8	K8_1	Test group	Soil inside a bronze *lei*	Near bronze
	K8_2	Test group	Soil inside a bronze *dakouzun*	Near bronze
	K8_3	Test group	Soil inside a bronze *lei*	Near bronze
	K8_4	Test group	Soil near a bronze *dakouzun*	Near bronze
	K8_5	Test group	Soil near a bronze ware	Near bronze
S_0	S_0	Control group B	Soil from the eastern slope outside pit K4, vertical depth 1 m	——

### 2.2 DNA extraction and amplicon generation

Total genomic DNA was extracted from 0.5 g soil samples using the FastDNA® Spin Kit for Soil (MP Biomedicals, LLC, USA) as recommended by the manufacturer’s instructions. The concentration and purification of DNA samples were detected with NanoDrop 2000 (Thermo Fisher Scientific Inc., USA). The integrity of DNA was examined using 1% agarose gel electrophoresis with the voltage of 5 V/cm for 20 min by the electrophoresis system JY-600C (Beijing Junyi Electrophoresis Co., Ltd., China).

Subsequently, PCR amplification was conducted using ABI GeneAmp® 9700 (Thermo Fisher Scientific Inc., USA). The primers of bacteria were designed based on the V4 hypervariable regions of 16S rRNA with 515F 5′-GTGYCAGCMGCCGCGGTAA-3′ and 806R 5′-GGACTACNVGGGTWTCTAAT-3′(Peiffer et al., 2013). The primers of fungi were amplified with the rRNA ITS1 hypervariable regions of ITS1F 5′-CTTGGTCATTTAGAGGAAGTAA-3′ and ITS2R 5′-GCTGCGTTCTTCATCGATGC-3′(He et al., 2021). After quality check of amplicons employing 2% agarose gel, the purification was conducted with the AxyPrep DNA Gel Extraction Kit (Axygen Scientific Inc., USA) according to the manufacturer’s instructions. The amplicons were quantified using Quantus™ Fluorometer (Promega Co., USA) for subsequent sequencing.

### 2.3 Library preparation and sequencing

The DNA libraries were established utilizing the NEXTFLEX Rapid DNA-Seq Kit (BIOO Scientific Co., USA) in accordance with the manufacturer’s instructions. The purified amplicons were pooling in equimolar amounts, and then, the DNA libraries were validated by the Agilent Fragment Analyzer 5400 (Agilent Technologies, Inc., USA) with the approximate size 450 bp and 480 bp for bacteria and fungi, respectively. After passing the test, sequencing was performed on an Illumina MiSeq PE300 platform (Illumina, Inc., USA) at Majorbio Bio-Pharm Technology Co., Ltd. (Shanghai, China).

### 2.4 Data processing

The raw sequencing files were quality-filtered and merged by fastp (version 0.19.6) (Chen et al., 2018) and FLASH (version 1.2.11) (Magoč and Salzberg, 2011), respectively, to select high quality data and remove the adapters. Reads were excluded with the score < 20. The optimized sequences were clustered into operational taxonomic units (OTUs) employing UPARSE (version 7.1) (Edgar, 2013) at the 97% sequence similarity (Li et al., 2017).

The most abundant sequence of each OTU was selected as a representative sequence, which was annotated the taxonomy via RDP Classifier (version 2.13). The bacterial sequences were submitted to the SILVA 138/16 s rDNA database^1^ with the confidence threshold of 70%. Similarly, the fungal sequences were analyzed at the same confidence threshold against the UNITE 8.0/its_fungi database.^2^

### 2.5 Statistical analysis

The bioinformatic analysis of all samples was carried out via the Majorbio Cloud platform.^3^ Alpha diversity (*α*-diversity, diversity within-sample) of microbiome was assessed by Mothur (version 1.30.2) with the indices of the observed richness (Sobs), the Chao1 estimator (Chao 1), the Shannon diversity index (Shannon), the Simpson diversity index (Simpson), and Pielou evenness index (Pielou_e). The Kruskal–Wallis *H*-test was performed to examine the significant differences between the different samples (*p < 0.05; **p < 0.01; ***p < 0.001). The beta diversity (*β*-diversity, similarity and difference between groups of samples) was visualized with principal co-ordinate analysis (PCoA) based on the Bray–Curtis distance via the vegan package in R3.3.1. The differences in microbial taxa of groups were performed by stats package in R3.3.1 and scipy package in python v1.0.0.

## 3 Results

### 3.1 Evaluation of microbial sequencing data

In total, 3,922,031 bacterial sequences and 4,655,448 fungal sequences were obtained from high-throughput sequencing for analysis after verifying, with the average length 256 bp of former and 240 bp of latter. Subsequently, the low-abundance OTUs with <10 copies in all samples were filtered to improve the reliability of microbial composition estimates (Bokulich et al., 2013; Nikodemova et al., 2023). Meanwhile, the number of effective sequences from all samples was rarefied to the number of the sample with minimum sequences, to minimize the effect of sequencing depth on the subsequent bioinformatics analysis.

To ensure the sequencing quantity and depth were sufficient and representative, the rarefaction curves of bacteria and fungi are given in Supplementary Figure S1, which tended to flatten out, suggesting that the sequencing data were able to comprehensively and truthfully reflect the microbial composition and community. Meanwhile, the Good’ s coverage (Supplementary Figures S1E,F) over 99.3% also indicated the sequencing data of all samples were enough for the follow-up analysis.

### 3.2 Alpha diversity of microbiome

The *α*-diversity of microbiome was characterized with Sobs, Chao 1, Shannon, Simpson, and Pielou_e indices, to assess the community richness, diversity, and evenness. The larger the values of Sobs and Chao 1, the higher the community richness. The larger the Shannon value and the smaller the Simpson value, the higher the community diversity. Similarly, the closer the Pielou_e value is to 1, the higher the species evenness. As shown in Table 2, the Sobs and Shannon indices of control group S_0 were largest for both bacteria and fungi, which meant the outside raw soil samples had the highest community richness and diversity, while the Pielou_e index on fungi of control group S_0 was smallest, meaning the lowest community evenness. As for the test groups, group K4 indicated the best biodiversity. The order of bacterial richness, diversity, and evenness in the lower of artifact layer was K4 > K8 > K3, while fungi was different with the order of K4 > K3 > K8.

**Table 2:** α-diversity index of soil samples.

Sacrificial pit	Name	Bacteria					Fungi				
		Sobs	Chao 1	Shannon	Simpson	Pielou_e	sobs	Chao 1	Shannon	Simpson	Pielou_e
K3_0	K3_0	620	674.52	4.75	0.03	0.74	18	18.000	2.338	0.128	0.81
K3	K3_1	281	335.38	3.05	0.10	0.54	42	43.200	1.909	0.217	0.51
	K3_2	443	563.01	3.57	0.07	0.59	34	38.667	1.253	0.412	0.36
	K3_3	252	287.28	2.82	0.13	0.51	40	39.667	2.553	0.134	0.71
	K3_4	236	255.83	2.74	0.21	0.50	31	31.333	2.075	0.224	0.60
	K3_5	320	376.94	3.60	0.06	0.62	22	23.667	1.810	0.308	0.59
Average of K3	/	306	363.69	3.15	0.11	0.55	34	35.307	1.920	0.259	0.55
K4_0	K4_0	1,063	1160.70	5.19	0.02	0.74	23	23.667	1.845	0.273	0.58
K4	K4_1	875	1028.27	4.88	0.02	0.72	14	13.667	1.776	0.237	0.68
	K4_2	487	591.53	3.87	0.05	0.63	44	44.333	2.263	0.167	0.60
	K4_3	397	514.33	3.55	0.06	0.59	70	71.222	2.616	0.114	0.62
	K4_4	332	394.45	3.14	0.10	0.54	42	42.500	2.303	0.165	0.62
	K4_5	622	696.97	4.34	0.04	0.67	19	19.333	1.055	0.566	0.35
Average of K4	/	543	645.11	3.96	0.06	0.63	38	38.211	2.003	0.250	0.58
K8_0	K8_0	414	480.97	3.06	0.12	0.51	30	30.000	1.831	0.290	0.54
K8	K8_1	457	548.47	3.58	0.09	0.58	17	17.000	2.189	0.188	0.78
	K8_2	741	906.49	4.39	0.04	0.66	39	38.667	2.928	0.090	0.80
	K8_3	286	347.91	2.48	0.23	0.44	27	27.000	1.104	0.498	0.34
	K8_4	396	487.84	3.59	0.09	0.60	37	37.667	1.240	0.504	0.33
	K8_5	312	345.94	3.57	0.06	0.62	19	18.667	1.155	0.555	0.39
Average of K8	/	439	527.33	3.52	0.10	0.58	28	27.800	1.723	0.367	0.53
Outside pits	S-0	1767	1911.62	5.92	0.01	0.79	267	275.063	2.656	0.290	0.47

All the values in the table are averages.

**Figure 3:**
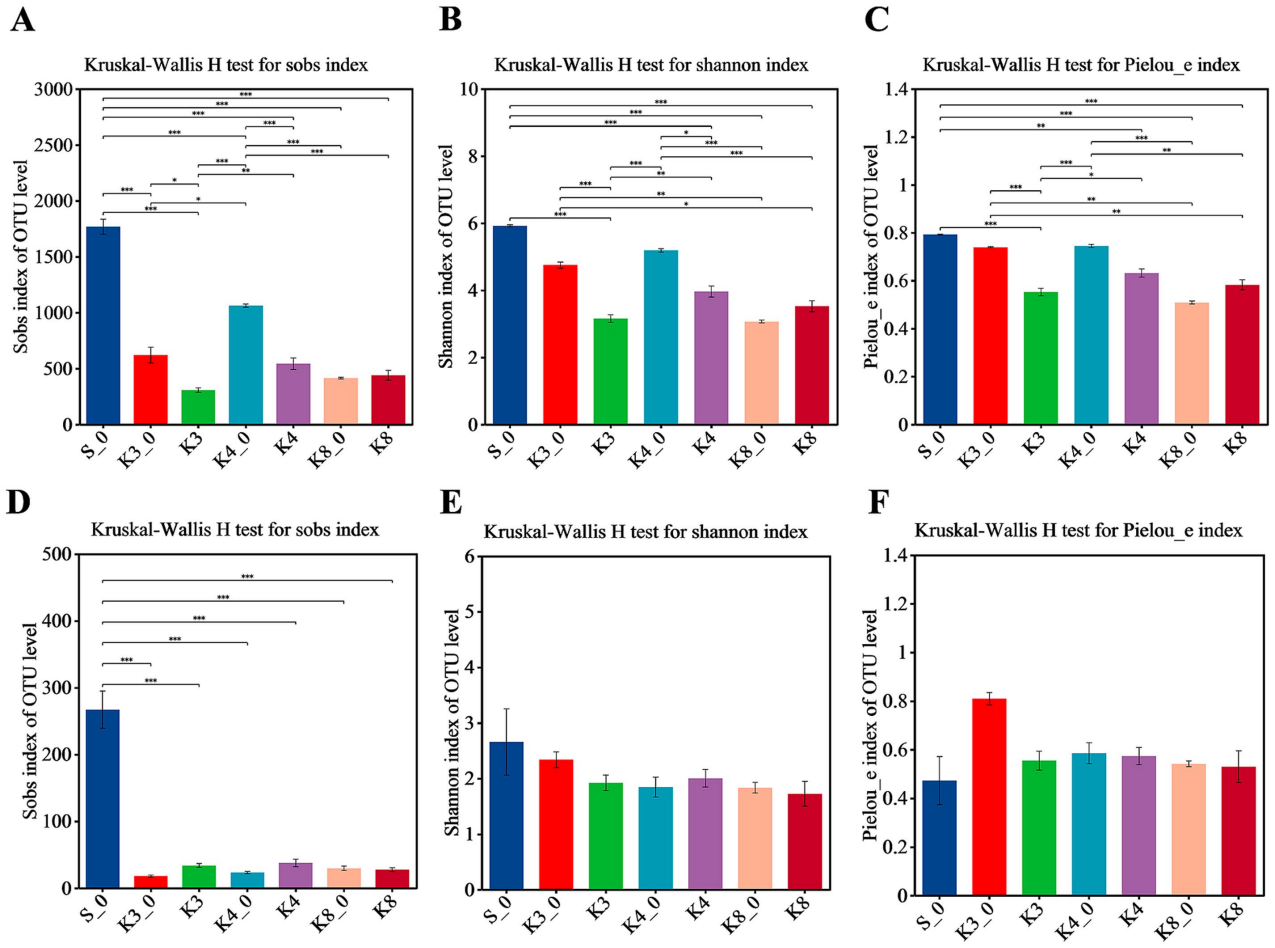
*α*-diversity shown by Sobs, Shannon, and Pielou_e. **(A–C)** Bacteria. **(D–F)** Fungi.

In terms of bacteria (Figure 3), almost all *α*-diversity indices displayed a highly significant difference (*p* < 0.001) between group S_0 and groups K3, K4, and K8. Group K3_0 and group K4_0 also indicated a difference (*p* < 0.05) or a highly significant difference (p < 0.001) in Sobs, Shannon, and Pielous_e indices from the group K3 and group K4, respectively. Furthermore, group K3 and group K4 also shown the statistical difference (*p* < 0.05) on Sobs, Shannon, and Pielous_e indices. The results demonstrated that the raw soil samples outside sacrificial pits (control group B), the raw soil samples inside sacrificial pits (control group A), and the test groups exhibited statistical difference in bacterial richness, diversity, and evenness, following the order: control group B > control group A > test group. Referring to fungi, the Shannon and Pielou_e indices did not perform the statistical difference, which meant the fungal diversity and evenness of all samples were closed, but the Sobs index of control group S_0 also shown the highly significant differences (*p* < 0.001) from other groups.

It was clearly observed that the statistical difference in α-diversity of bacteria was much higher than that of fungi. In addition, the statistical difference between different samples from the same sacrificial pit in bacteria and fungi was still remained (Supplementary Figures S2, S3), especially for control groups K3_0, K4_0, and K8_0 and the test samples from corresponding sacrificial pits. The results probably were caused by the different sampling locations and the nearby artifacts in spite of the same sacrificial pit.

### 3.3 OTU characterization of microbiome

**Figure 4:**
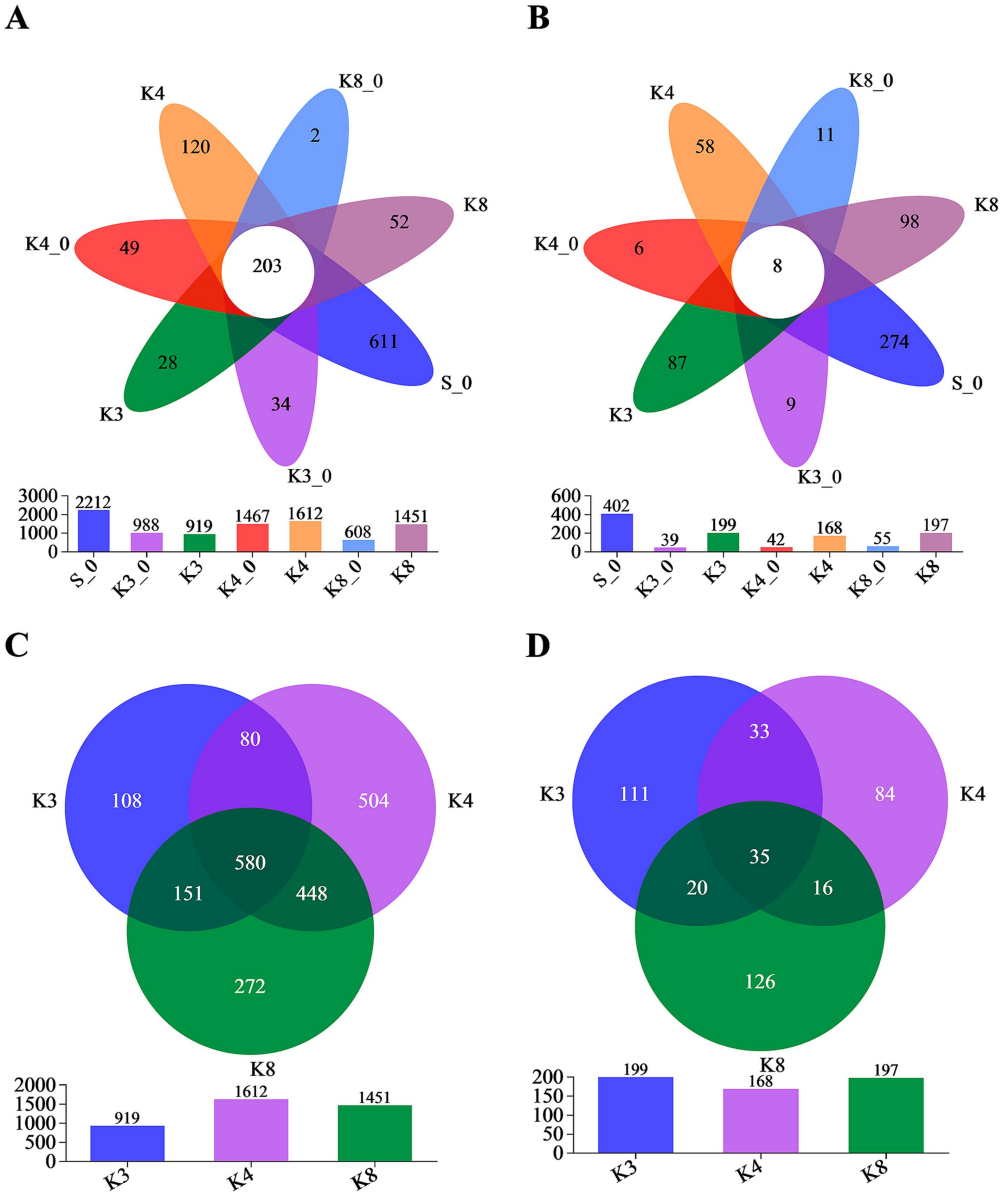
Venn diagram on the OTU level. **(A)** OTUs of bacteria in all groups. **(B)** OTUs of fungi in all groups. **(C)** OTUs of bacteria in groups K3, K4, and K8. **(D)** OTUs of fungi in groups K3, K4, and K8.

Through the trimmer and filtering methods, a total of 3,174 bacterial OTUs and 733 fungal OTUs were detected with high reliability and accuracy. According to Venn plot (Figure 4), there were 2,212 bacterial OTUs and 402 fungal OTUs in the control group S_0, which were much higher than other groups. In test groups, the order of the bacterial OTUs was following K4 > K8 > K3, and the order of the fungal OTUs was K3 > K8 > K4.

More importantly, Figure 4 also displays the number of unique (in side) or shared (in core) OTUs in different groups, which revealed the similarity and overlap across sacrificial pits. Only 203 bacterial OTUs and 8 fungal OTUs were common to all groups, while 580 bacterial OTUs and 35 fungal OTUs were shared in groups K3, K4, and K8, which indicated the test groups K3, K4, and K8 exhibited higher overlap with each other rather than with control groups. Simultaneously, there were also numerous OTUs shared between two of the sacrificial pits or only appeared in one pits, suggesting the heterogeneity of the test groups from different sacrificial pits on OTU level. The similar overlap and differences were observed as well in OTU results of individual samples (Supplementary Figure S4). Even though samples collected from the same sacrificial pit, the OTUs of different samples still showed differences. The results above probably indicated the impact of excavation location and buried artifacts on soil microbiome.

### 3.4 Identification and composition of microbial community

**Figure 5:**
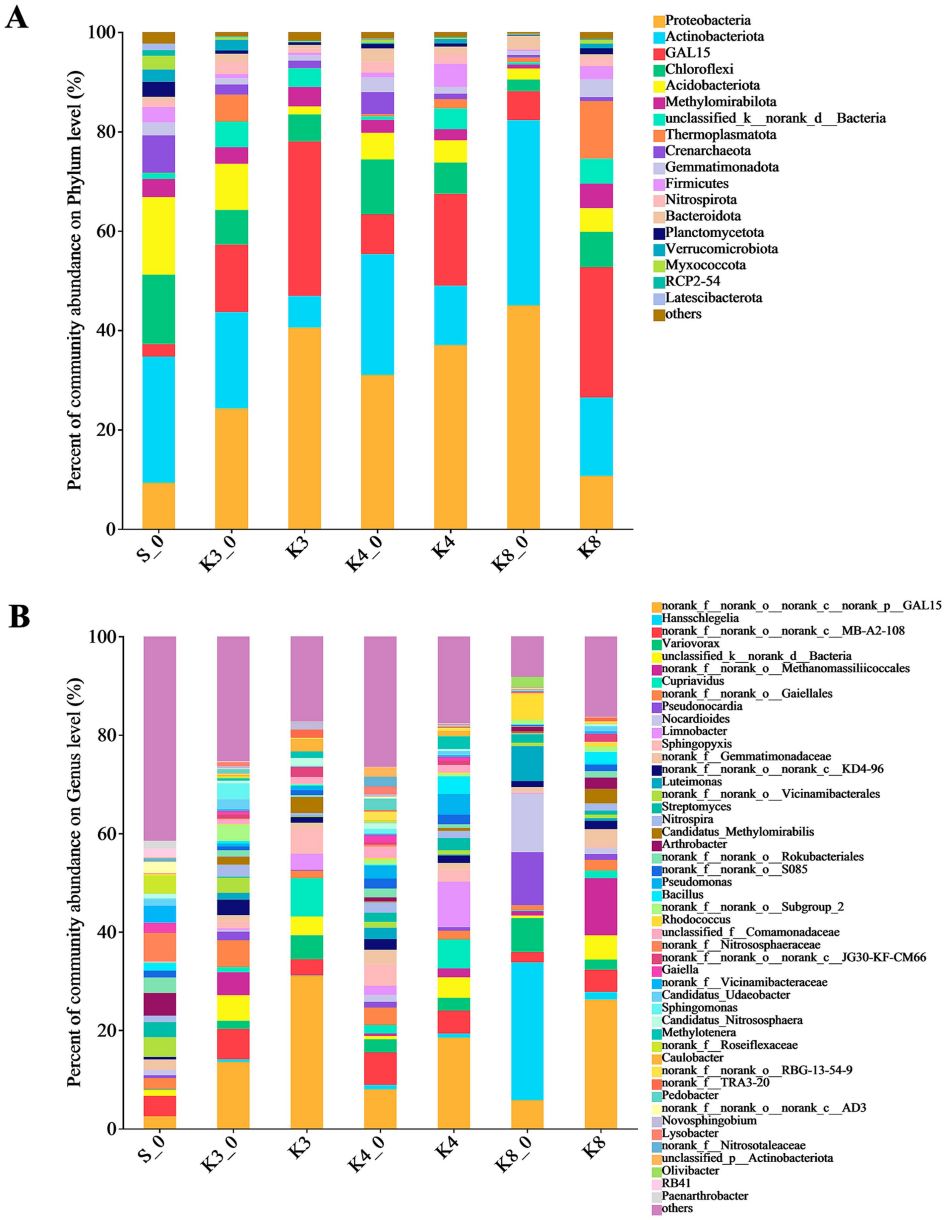
Microbial distribution of soil bacteria. **(A)** Phylum level. **(B)** Genus level.

To analyze the community composition and structure, the microbial OTUs were further classified. The bacterial OTUs were divided into 42 phyla, 405 families, and 667 genera. As shown in Figure 5, there were 18 dominant bacterial (relative abundance >1%) phyla in groups. The top bacterial (relative abundance >2%) phyla were Proteobacteria, Actinobacteriota, GAL15, Chloroflexi, Acidobacteriota, Methylomirabilota, unclassified_k__norank_d__Bacteria, Thermoplasmatota, Crenarchaeota, Gemmatimonadota, Firmicutes, and Nitrospirota, which were detected in all groups, and the combined abundance of these bacteria exceeded 80%. The phyla above were common to the samples of human archaeological remains. The results indicated that the test and control groups were similar at the phylum level in bacteria but differed in relative abundance.

The differences in bacterial community and composition among different pits were further noticed at the genus level. There were 65 dominant bacterial (relative abundance >1%) genera in samples. It should be noted that some norank and unclassified genera, such as genera *norank_f__norank_o__norank_c__norank_p__GAL15*, *unclassified_d__Bacteria*, and *norank_f__norank_o__Gaiellales*, were identified in all groups but lacking the accurate identification information. Thus, the bacteria only identified to order level were filtered out. The bacterial composition showed the evident differences between different groups. In group S_0, the top bacterial genera (relative abundance >2%) were genera *Arthrobacter* (4.65%), *Streptomyces* (3.07%), as well as genera *norank_f__Nitrososphaeraceae* (5.75%), *norank_f__Vicinamibacteraceae* (3.48%), *norank_f__Roseiflexaceae* (3.66%), and *norank_f__Gemmatimonadaceae* (2.04%). In group K3_0, the genera *Sphingomonas* (3.20%), *Nitrospira* (2.41%), and *Candidatus_Udaeobacter* (2.02%) were found. Meanwhile, there were six top bacterial genera including genera *Cupriavidus* (7.72%), *Sphingopyxis* (5.19%), Var*iovorax* (4.91%), *Limnobacter* (3.28%), *Candidatus_Methylomirabilis* (3.26%), and *Caulobacter* (2.56%) in group K3. Similarly, eight top bacterial genera including the genera *Limnobacter* (9.20%), *Cupriavidus* (5.86%), *Pseudomonas* (4.20%), *Bacillus* (3.55%), *Methylotenera* (2.63%), *Variovorax* (2.57%), *Sphingopyxis* (2.49%), and *Streptomyces* (2.53%) were detected in group K4. In group K4_0, the top bacterial genera were genera *Sphingopyxis* (4.42%), *norank_f__Gemmatimonadaceae* (2.93%), *Pseudomonas* (2.65%), *Variovorax* (2.63%), *Pedobacter* (2.37%), *Luteimonas* (2.32%), *unclassified_f__Comamonadaceae* (2.30%), and *Nitrospira* (2.13%). Obviously, group K3 and group K4 had more precise dominant bacterial genera with higher abundance than group K3_0 and group K4_0, respectively. As for group K8, the top bacterial genera were genera *Candidatus_Methylomirabilis* (2.95%), *Bacillus* (2.45%), *Arthrobacter* (2.29%), *Variovorax* (2.57%), and *norank_f__Gemmatimonadaceae* (3.60%), while group K8_0 included more bacterial genera, such as genera *Hansschlegelia*, *Nocardioides*, *Pseudonocardia*, *Luteimonas*, *Variovorax*, *Rhodococcus*, and *Olivibacter*, accounting for 28.01, 11.97, 10.78, 7.12, 6.88, 5.42, and 2.13%, respectively.

**Figure 6:**
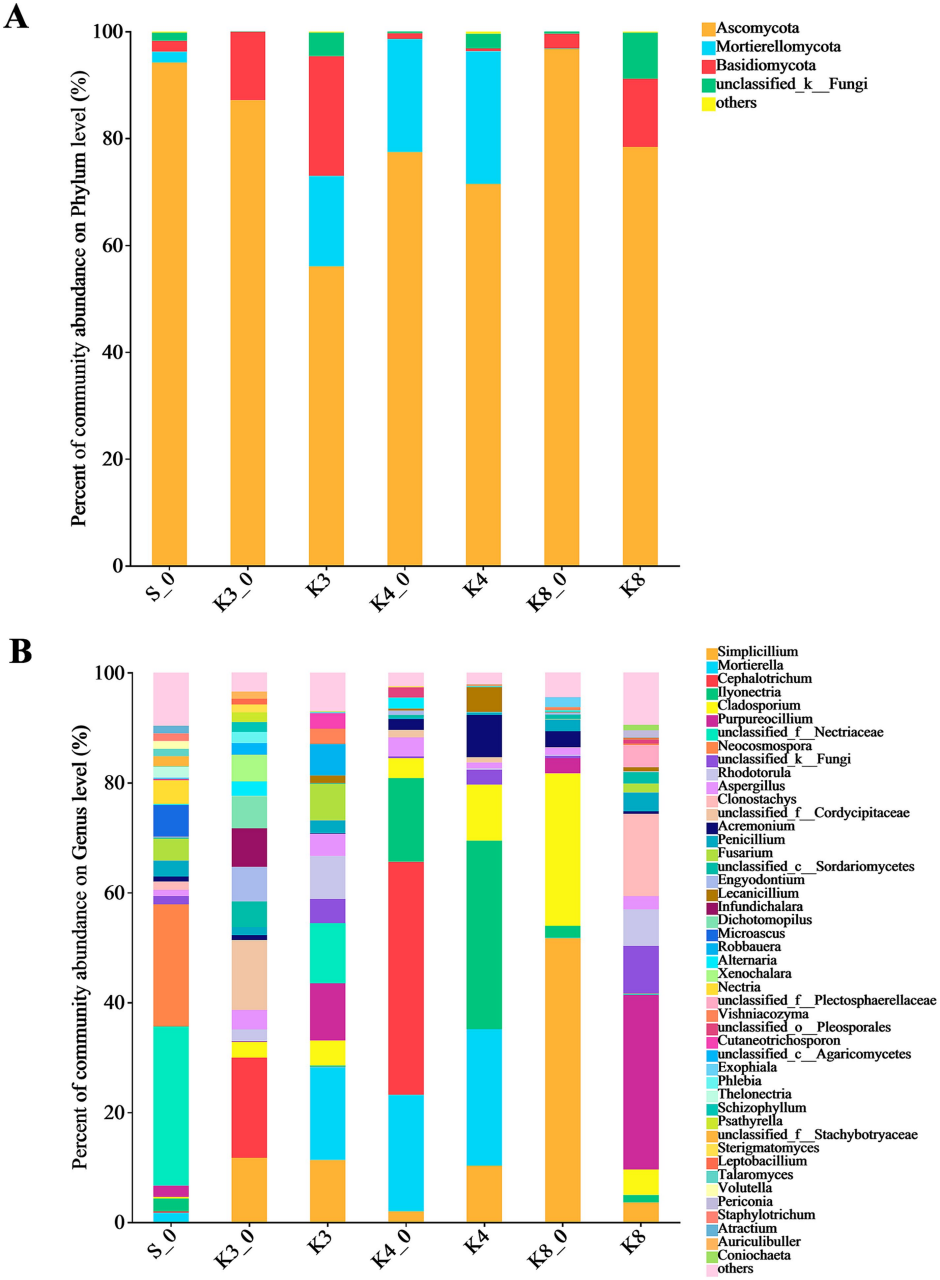
Microbial distribution of soil fungi. **(A)** Phylum level. **(B)** Genus level.

The fungal OTUs belonged to 12 phyla, 182 families, and 298 genera. There were only four dominant fungal phyla (relative abundance >1%) in groups, which included Ascomycota, Mortierellomycota, Basidiomycota, and an unclassified_k__Fungi (Figure 6). It is worth noting that Mortierellomycota was not detected in groups K3_0 and K8, although it was more abundant in groups K3 and K4. At the genus level, the number of dominant fungal genera (relative abundance >1%) was 46. Similar to the bacterial results, the fungal genera in groups displayed the remarkable differences between groups. In group S_0, the top fungal genera (relative abundance >2%) were genera *unclassified_f__Nectriaceae* (28.92%), *Neocosmospora* (22.23%), *Microascus* (5.76%), *Nectria* (4.31%), *Fusarium* (3.97%), *Penicillium* (2.91%), *Ilyonectria* (2.32%), *Purpureocillium* (2.06%), and *Thelonectria* (2.02%). The top fungal genera in group K3 were genera *Mortierella* (16.93%), *Simplicillium* (11.33%), *Purpureocillium* (10.40%), *unclassified_f__Nectriaceae* (10.96%), *Rhodotorula* (7.78%), *Fusarium* (6.61%), *Robbauera* (5.72%), *Cladosporium* (4.52%), *Aspergillus* (4.05%), *Cutaneotrichosporon* (2.80%), *Vishniacozyma* (2.79%), *and Penicillium* (2.35%), while the different fungi such as genera *Cephalotrichum* (18.19%), *unclassified_f__Cordycipitaceae* (12.71%), *Infundichalara* (7.00%), *Engyodontium* (6.28%), *Dichotomopilus* (5.87%), *Xenochalara* (4.85%), and *Alternaria* (2.68%) were found in group K3_0. In group K4, the top fungal genera were more concentrated in genera *Ilyonectria*, *Mortierella*, *Simplicillium*, *Cladosporium*, *Acremonium*, and *Lecanicillium*, with the relative abundance of 34.29, 24.87, 10.29, 10.20, 7.70, and 4.51%, respectively. The control group K4_0 shown the accordant fungi with group K4 in addition to genera *Cephalotrichum* (42.38%) and *Aspergillus* (3.45%). In group K8, genera *Purpureocillium* (31.76%), *Clonostachys* (14.96%), *Rhodotorula* (6.65%), *Cladosporium* (4.63%), *unclassified_f__Plectosphaerellaceae* (3.95%), *Simplicillium* (3.61%), *Penicillium* (3.45%), and *Aspergillus* (2.41%) were the top fungal genera. Apparently, the types and relative abundance of different fungi in group K3 and group K8 differed obviously from group K3_0 and group K8_0, respectively. In contrast, the groups K4 and K4_0 displayed a certain similarity in fungal community composition.

### 3.5 Beta diversity of microbiome

**Figure 7:**
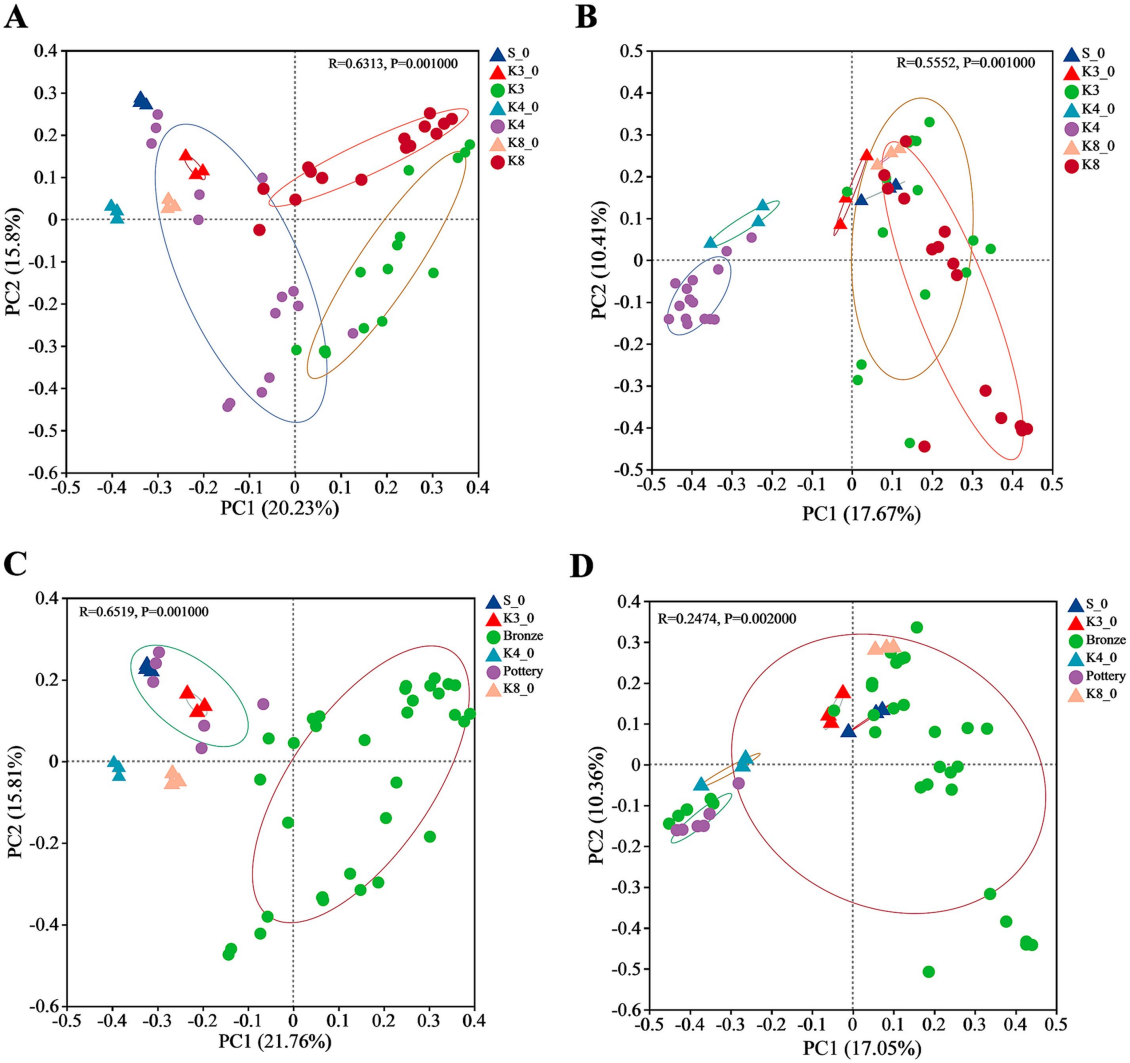
PCoA of bacteria and fungi in different groups. **(A)** Bacteria in all groups. **(B)** Fungi in all groups. **(C)** Bacterial in soil samples near bronzes and pottery sherds. **(D)** Fungi in soil samples near bronzes and pottery sherds.

The similarities and differences of bacterial and fungal communities were evaluated via clustering using Bray–Curtis distance-based principal co-ordinate analysis (PCoA) on the OTU level. The PCoA result of bacteria (Figure 7A) revealed that the sacrificial pits of K3, K4, and K8 shown significant difference in taxa biodiversity. Especially, group K4 exhibited the evident differences from groups K3 and K8. Group K4 also shown a dispersed distribution, which was closer to the control groups. As for fungi (Figure 7B), the group K4, as well as group K4_0, also performed significant difference with other groups, whereas it is hard to distinguish the samples of groups K3 and K8, suggesting that the fungal community between group K3 and group K8 was similar. The microbial distribution and structure were also performed by hierarchical clustering analysis (HCA, Supplementary Figures S5, S6), which were consistent with the PCoA results. Compared to group K4, group K3 and group K8 show higher similarity in bacteria and fungi communities and taxa biodiversity.

In addition, it should be noted that the location of samples and the material of artifacts around the samples, including pottery and bronze, also affect the microbial diversity. As shown in Figures 7C,D, the samples near pottery and bronze displayed the differences in bacterial biodiversity, but the distinction in fungi was not observed.

### 3.6 Differences in microbial taxa and composition

**Figure 8:**
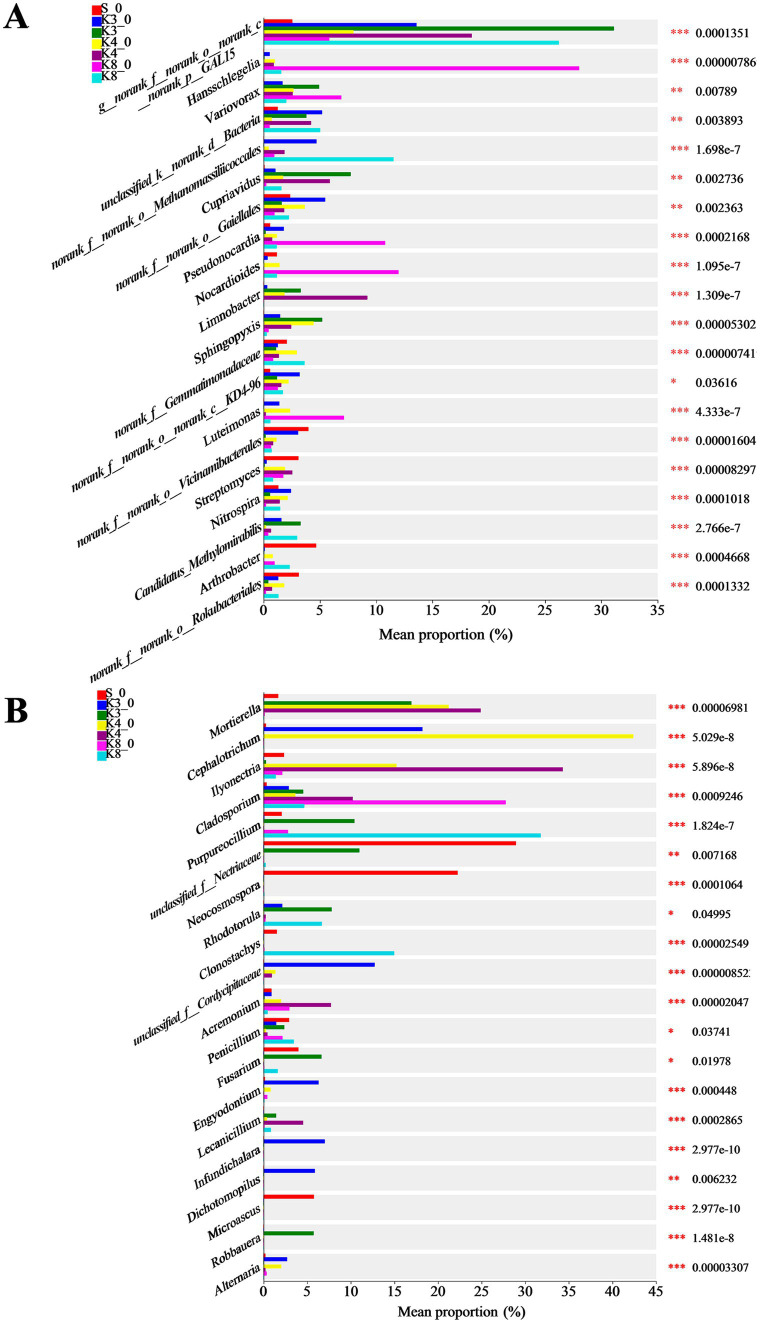
Differences in microbial taxa in all groups. **(A)** Bacteria. **(B)** Fungi.

Based on the microbial results above, the differences in community and composition were further investigated. The top 20 bacterial and fungal genera are exhibited in Figure 8. As for bacteria, there were 15 genera manifesting the highly significant difference (*p* < 0.001) in different groups. The genus *norank_f__norank_o__norank_c__norank_p__GAL15* was dominant in groups K3, K4, and K8. The genera *Candidatus_Methylomirabilis*, *Sphingopyxis*, and *Cupriavidus* were dominant in group K3. The genera *Limnobacter*, *Pseudomonas*, and *Bacillus* were more abundant in group K4. The genera *norank_f__Gemmatimonadaceae* and *Candidatus_Methylomirabilis* were more abundant in group K8.

Similarly, 15 genera of fungi performed the highly significant difference (*p* < 0.001). The genera *Robbauera*, *Fusarium*, and *Rhodotorula* were more abundant in group K3. The genera *Ilyonectria* and *Mortierella* were dominant in group K4. In group K8, the abundance of genera *Purpureocillium* and *Clonostachys* increased markedly. From the results of difference analysis, it could be seen that there were statistical difference in the abundance of different genera in each sacrificial pit.

## 4 Discussion

Combining the results of *α*-diversity and OTU characteristics, it has been known that the group S_0 shown the superior biodiversity than test groups in sacrificial pits K3, K4, and K8 and also exhibited the significant statistical difference in community richness, evenness, and diversity simultaneously. Moreover, the microbial composition of group S_0 exhibited a richer genera, which might be consistent with the fact that the soil that has not been disturbed by humans. In contrast, although there were differences in biodiversity between sacrificial pits K3, K4, and K8, the differences were smaller than those between group S_0. The more direct result was that the shared OTUs among groups K3, K4, and K8 increased significantly (Figures 5C,D). However, in terms of microbial community and structure, groups K3 and K8 were more similar and quite different from group K4 according to the PCoA results. Further comparing the microbiome in the same sacrificial pit, it could be found that the soil samples located in different artifacts exhibited the different microbial composition and structure with the relatively dispersed PCoA plots. In sacrificial pits K3, K4, and K8, we found that group K4 has shown the most homogeneous microbial community with the highest value of community evenness and most concentrated PCoA plots. An interesting phenomenon was observed in fungi that the order of fungal diversity was K4 > K3 > K8 based on the α-diversity, but with the different OTUs order of K3 > K8 > K4, which could be explained by the community evenness index of Pielou_e (Table 1). The Pielou_e values of fungi in group K4 were closer except for sample K4_5, also with the highest average of 0.58, resulting in the most shared fungal OTUs in group K4. The results demonstrated that the buried environment and the excavation location were affected microbial diversity. Even within the same sacrificial pit, the microbial composition and diversity were not exactly the same.

### 4.1 Microbial function and feature in sacrificial pits

A great number of bacteria and fungi exist in soil (Janssen et al., 2002). It is one of the most crucial aim to identify and compare the microbial community and composition of soil in different sacrificial pits. In recent years, several investigations have been conducted in the human archaeological remains (Philips et al., 2017). Some of the most common soil bacteria are considered to be Firmicutes, Actinobacteriota, Actinobacteria, Proteobacteria, Acidobacteriota, Methylomirabilota, Chloroflexi, Bacteroidota, and Nitrospirota (Kazarina et al., 2019; Wei et al., 2022). The fungal phyla of Ascomycota and Basidiomycota are dominated in human archaeological remains, followed by Mortierellomycota, Chytridiomycota, or Zygomycota (Margesin et al., 2016; Siles et al., 2018; Voyron et al., 2022). All bacterial and fungal communities collectively reflect the serious impact of ancient anthropogenic activities on soil environment.

In our results of bacteria, Proteobacteria were the most widespread phylum in the test groups, followed by Actinobacteriota, GAL15, Chloroflexi, Acidobacteriota, etc. (Figure 5). Proteobacteria are the largest and phenotypically most diverse phylogenetic lineage, which contain strictly anaerobic and aerobic species, facultative aerobes, and microaerophiles to participate the carbon, nitrogen, and sulfur cycles on Earth (Kersters et al., 2006). In Sanxingdui sacrificial pits, Proteobacteria were dominated via Alphaproteobacteria and Gammaproteobacteria. The former included the genera *Caulobacter*, *Hansschlegelia*, *Sphingomonas*, and *Sphingopyxis*. The latter contained genera *Cupriavidus*, *Limnobacter*, Var*iovorax*, *Methylotenera*, *Pseudomonas*, and *Luteimonas*. Among them, genus *Hansschlegelia*, the most abundant in group K8_0, represented a novel lineage of autotrophic methanol-utilizing bacteria as the carbon and energy source in plant and soil (Ivanova et al., 2007; Wen et al., 2011). Genus *Sphingopyxis* has been reported in catacombs (Alias-Villegas et al., 2013), which is also widespread in diverse ecological environments, including soil, sediment, water, and heavy metal contaminated sites (Sharma et al., 2021). It has attracted considerable attention not only for excellent ability to survive under extreme environments but also for the ability to degrade organic matter. Therefore, although the content of copper ion was high in the soil of sacrificial pits, genus *Sphingopyxis* was still found dominantly in K3, K4, and K4_0, probably indicating the presence and biodegradation of organic matter. Genus *Limnobacter* is an obligately aerobic and grew on various organic substrates, but it has been designated chemolithoheterotrophs, which is able to grow by oxidation of thiosulfate to sulfate (Spring et al., 2001). The capabilities might indicate the presence of sulfide or degradation of organic matter in sacrificial pits. Genus *Variovorax* generally inhabits soil and water and plays an important role in the regulation of plant growth and development (Finkel et al., 2020). Meanwhile, the genus is found in the ancient apple seeds (Milanesi et al., 2019) and decaying woods (Ghio et al., 2012). Genus *Variovorax*, hence, is considered as a possible source of hydrolytic enzymes to degrade cellulose. Moreover, it should be noted that the genus is often detected after bushfire (Smith et al., 2008). Therefore, the presence in all groups except group S_0 might indicate the impact of high temperature processes in the ash layer on bacteria.

Actinobacteriota, the second abundant bacterial phylum in our results, are generally distributed across both terrestrial and aquatic environments, as well as in the microbiome of higher eukaryotes. The phylum shows the unrivalled metabolic versatility, producing highly diverse bioactive natural products, but it must be noted that there were lots of unclassified genera with high abundance in Actinobacteriota in our results, such as genera *g__norank_f__norank_o__norank_c__MB-A2-108* and *g__norank_f__norank_o__Gaiellales*. Therefore, it is difficult to analyze their properties and functions in the ecosystem. Furthermore, the genera *Streptomyces*, *Rhodococcus*, *Nocardioides*, and *Pseudonocardia* were identified in sacrificial pits, which are found in ancient wall painting, ancient seeds, wooden shipwreck, tomb, or cave (Stakhov et al., 2008; Juanjuan et al., 2016; Liu et al., 2018; Sakr et al., 2020). Among them, genus *Streptomyces* can cause the deterioration of complex natural polymers via producing a great deal of enzymes (Sakr et al., 2020). The genus was dominant in groups K4 and S_0, probably indicating the presence of natural polymers. The phylum Methylomirabilota plays a key role in global carbon and nitrogen cycle as the methane oxidizing bacteria. The genus *Candidatus_Methylomirabilis* participates in the cycle by oxygenic denitrification and aerobic methane oxidation pathways (Sun et al., 2024). The dominant abundance in groups K3 and K8 indicated that a certain amount of organic matter probably was present in sacrificial pits.

In the part of fungi, Ascomycota were the most abundant fungal phylum, with the genera *Cladosporium*, *Aspergillus*, *Penicillium*, *Simplicillium*, *Purpureocillium*, *Lecanicillium*, and *Acremonium*. Among them, the genera *Cladosporium* and *Simplicillium*, belonging to family Cordycipitaceae, were dominant in all groups expect for group S_0, suggesting the effect of human burial behavior and buried artifacts. The genus *Cladosporium* is isolated in ancient wall painting and beeswax drops on ancient papers (He et al., 2021; Pavlović et al., 2022), showing the proteolytic and lipolytic abilities in the process of organic degradation. In addition, previous studies have indicated that the genus *Cladosporium* is capable of hydrolyzing cellulose by the production of cellulases (Leung et al., 2016; Li et al., 2019). The genus *Simplicillium* is identified on the polychrome pottery figurines (Zhang et al., 2023), resulting in the fading of the blue (azurite) and green (malachite) pigments through the absorption of copper (II) ion. Moreover, the genus has been proved to degrade tempera paintings and artifacts made with dammar varnish and rock-art paintings (Jurado et al., 2008). So genus *Simplicillium* is destructive to both organic and inorganic materials. The genera *Lecanicillium* and *Acremonium* belong to the order Hypocreales. Genus *Lecanicillium* contains some entomopathogenic species, and some are capable to degrade organic compounds (Fenice, 2016; Pozdnyakova et al., 2019). For instance, *Lecanicillium muscarium* is a potent producer of extracellular cold-resistant, chitin-hydrolyzing enzymes for the degradation of chitin-rich materials (Fenice, 2016), but the degradative properties of the genus *Lecanicillium* have been poorly studied. The genus *Acremonium* is the environmental saprophytes in soil and decaying plant material and is able to secrete a lot of cellulolytic enzymes with high activity (Amiri et al., 2011; Hu et al., 2015). The genus is found in ancient wall paintings (He et al., 2021), and the dominant proportion in group K4 probably implied the degradation of cellulose organic matter.

The genera *Robbauera*, *Rhodotorula*, *Vishniacozyma*, and *Cutaneotrichosporon*, belonging to Basidiomycota, were found in K3 and K8, especially in group K3 with the dominant abundance. Among them, genus *Vishniacozyma* has been identified as the core composition during the spontaneous fermentation of ice wine, probably relating to the fermentation process (Chen et al., 2020). As the pigmented yeast, genus *Rhodotorula* is strict aerobic yeasts with peculiarly metabolic characteristics. The genus is very important for food industries because it can produce glycogen, lipids, and carotenoid pigments during the growth phase (Hernández-Almanza et al., 2014), but it should be noted that the genus does not ferment carbohydrates (Dworecka-Kaszak and Kizerwetter-Świda, 2011). In addition, genus *Mortierella*, belonging to Mortierellomycota, was the core part in groups K3, K4, and K4_0. The genus is the widespread filamentous fungi that can decompose various organic compounds such as cellulose, hemicellulose, and chitin (Ozimek and Hanaka, 2021).

### 4.2 The biocorrosion of bronzes in sacrificial pits

The problem of biocorrosion could not be ignored during thousands of years of burial. Considering that the bronzes were the most unearthed artifacts, the biocorrosion (also known as microbiologically influenced corrosion, MIC) of bronzes was the major concern. Some microbial taxa known to corrode metal material or solubilizing metals from metal-bearing substrates were found. As for bacteria, the genus *Pseudomonas* is repeatedly identified in archaeological remains and cultural heritage (Li et al., 2018; Siles et al., 2018; He et al., 2021; Pavlović et al., 2022). Numerous studies have shown that the genus *Pseudomonas* contains nitrogen-fixing bacteria and nitrate-reducing bacteria (NRB) (Dou et al., 2022; Wu et al., 2023). The NRB can reduce nitrate under biocatalysis by transporting the electrons into the cell cytoplasm and further cause the biocorrosion. The genus *Arthrobacter* is an aerobic culturable bacteria in soil and is also thought to be able to decompose various organic matter (Yao et al., 2015). More importantly, the genus also contained some species with excellent tolerance to excess heavy metal, which formed biofilms and colonized on the surface of copper, resulting in the pitting corrosion (Swaroop et al., 2016). The Var*iovorax* sp. has also been isolated from bacterial biofilms of copper pipes, probably leading to the biodeterioration (Reyes et al., 2008), which might be the reason why the genus was significant abundance in all sacrificial pits and almost absent in control group S_0. In addition, the genus *Cupriavidus* also shows the prominent resistance to high concentrations of copper ion, and its growth initiation is strongly stimulated by copper in some instances (Makkar and Casida, 1987; Gillan et al., 2017). So it was reasonable that genus *Cupriavidus* was present in groups K3, K4, and K8 in spite of the high concentration of copper ion from bronzes.

Many fungal genera are also capable of corrosion metals. Recent study has demonstrated that the conidia and hyphae of some fungi adhere to the surface of the metal. They penetrate into the surface layer of metal and go deep into the bulk, finally causing corrosion damage with pitting, ulcers, and cavities (Belov et al., 2023). The genus *Penicillium* was found in all groups and dominant in groups K3 and K8, which has been proven to be able to solubilize several metals including zinc, stannum, aluminum, plumbum, and copper (Siegel et al., 1983). The genus *Aspergillus* exhibits the marked tolerance to copper and also shows the evident biocorrosion of copper metal through proton-and ligand-mediated dissolution mechanisms, causing obvious mass loss and surface etching (Zhao et al., 2020). This was a good explanation for why the genus was the dominant fungi in sacrificial pits. Genus *Rhodotorula*, dominant in groups K3 and K8, unexpectedly, was also identified in biofilms that causes MIC in copper pipes, probably meaning that the genus was involved in the corrosion of bronzes (Reyes et al., 2008) in sacrificial pits. In addition, the genus *Cladosporium* was also involved in the biodeterioration of metal (Iverson, 1987).

As we all know, the MIC process of metals is related to the formation of microbial biofilms, which can form on any metal surface. The bacterial and fungal colonization in biofilms can modify the localized environment at the metal surface and cause corrosion. Generally, there are two typical MIC processes, namely, the extracellular electron transfer MIC (EET-MIC) and metabolite MIC (M-MIC) (Dou et al., 2022). The former mainly utilizes EET to transport the electrons in the corrosion process of sulfate-reducing bacteria (SRB) and NRB because metal oxidation occurs extracellularly while sulfate or nitrate reduction occurs intracellularly with biocatalysis (Di, 2022). The genus *Pseudomonas* and genus *Arthrobacter* are usually considered to corrode the copper by EET-MIC. For example, *Pseudomonas aeruginosa* utilizes copper as an electron donor, and the terminal electron acceptor is nitrate, causing and accelerating the corrosion of bronzes (Dou et al., 2022). The *Arthrobacter sulfureus* is electrochemically active and is able to transfer electron through the membrane-associated C-cytochromes or through conductive pili (Swaroop et al., 2016). As for the other biocorrosion mechanism, the M-MIC often is caused by corrosive metabolites such as organic acids secreted by bacteria and fungi. The genus Var*iovorax* in biofilm may destroy copper by releasing acid metabolites. Moreover, more and more research studies (Rao et al., 2002; Anjum et al., 2009; Amiri et al., 2011) also demonstrate that the fungi such as genera *Penicillium* and *Aspergillus* can generate varieties of organic acid metabolites including citric acid, lactic acid, oxalic acid, malic acid, and gluconic acid to dissolve and leach metals effectively, especially copper.

### 4.3 The effect of burial activity and artifact material on microbiome

By comparing the results of microbial diversity of different groups, it was clear to observe that the microbial composition and structure of raw soil samples exhibited an significant difference from the test samples in sacrificial pits K3, K4, and K8, meaning the microbial community could be modified in the archaeological site because of the buried man-made structure and artifacts. The results were consistent with previous studies (Junco et al., 1992). Compared to the control group S_0, there were many dominant bacteria and fungi, such as genera *Caulobacter*, *Sphingomonas*, *Sphingopyxis*, *Limnobacter*, *Cladosporium*, *Acremonium*, *Simplicillium*, and *Mortierella* in test samples in sacrificial pits. Some of them were related to the degradation of organic matter, indicating that there might be a certain amount of organic matter ever buried in the sacrificial pits, such as proteins, lipids, and carbohydrates. Unfortunately, as for organic residues, only some textile traces were found on the surface of the bronzes. Although a large amount of ivory was preserved, the collagen in it was almost completely degraded (Honglin et al., 2022). More investigations are needed to identify and demonstrate the presence of different organics in sacrificial pits in the further. The other dominant bacterial genera *Pseudomonas*, *Arthrobacter*, *and Variovorax* and fungal genera *Aspergillus* and *Penicillium* in sacrificial pits were in connection with the biocorrosion of bronzes, also revealing the impact of the buried bronzes on microbiome. In addition, the phylum GAL15 was the primary bacteria in all sacrificial pits. The abundance of bacteria GAL15 in different groups followed the distribution pattern: raw soil outside pits (group S_0) < raw soil in pits (group K3_0, K4_0, and K8_0) < test soil in artifact layer (groups K3, K4, and K8). These results demonstrated that the abundance of phylum GAL15 might be related to the buried artifacts, especially for the bronzes in sacrificial pits, which were consistent with previous findings in ivory layer of Sanxingdui site (Sun et al., 2024). Unfortunately, none of family or genus was matched in this phylum to in-deep analysis.

In addition, the proportion of buried artifacts of different materials also affected the microbial community. The results of *β*-diversity (Figure 7) indicated that the bacterial and fungal communities of group K3 and group K8 were much closer, and both differed significantly from those of group K4. This phenomenon might result from the material of the buried artifacts. Based on the excavation brief of sacrificial area of Sanxingdui site (Honglin et al., 2022), there are 2,686 numbered artifacts in K3, of which bronzes are the largest in number at 1,171, accounting for 43.60%. As for K8, there are 5,210 artifacts unearthed as the publication of the brief report, with 3,822 bronzes, accounting for 73.36%. Furthermore, the brief excavation report of K4 has been published separately (Danyang et al., 2024), which records the exact number of artifacts in artifact layer. There are 1,118 artifacts found in artifact layer of K4, with 468 pottery sherds and 348 bronzes. The proportions of pottery and bronzes were 41.86 and 31.13%, respectively. The large number of bronzes in K3 and K8, therefore, had a significant impact on the microbial composition and structure, causing the evidence differences from K4. Recent studies have revealed that the copper contents in soil near bronzes in sacrificial pits of Sanxingdui site increased several times (Jianguo et al., 2023). Many metals are essential for life, such as copper, sodium, kalium, zinc, and iron, while all metals can exert toxicity when presented above certain threshold concentrations (Gadd, 2010). The high content of copper ion in soil could inhibit the growth of certain microorganisms, which led to the significant difference of bacterial and fungal communities between different sacrificial pits, also between the sacrificial pits and soil outside pits. The PCoA results of bronzes and pottery sherds (Figures 7C,D) suggested the impact of copper ion was more obvious on bacteria than fungi, with the more concentrated distribution of the same type samples. Meanwhile, the bacterial community of all raw soils either inside pits or outside pits was closer to group K4, because of the lower proportion of the bronzes in K4.

## 5 Conclusion

In summary, the bacteria were classified into 42 phyla, 405 families, 667 genera, and 3,174 OTUs, while the fungi were annotated into 12 phyla, 182 families, 298 genera, and 733 OTUs. Some microbes associated with the deterioration of organics and bronzes were found in sacrificial pits, indicating that biodeterioration was the important cause for the degradation and corrosion of sacrificial offering or buried artifacts. Simultaneously, the analysis of the microbial profiles of the soil samples from Sanxingdui site indicated the significant differences in microbial diversity and community between raw soil and soil around artifacts in sacrificial pits. The differences of *α*-diversity, OTU characterization, microbial composition, and community between raw soil groups and test groups revealed the huge impact of human sacrificial and burial activities on microbiome. The microbial diversity in artifact layer in the sacrificial pits decreased, while the bacteria and fungi resistant to heavy metals and degrading organic matter increased. The microbial diversity analysis, therefore, may be an available method to speculate and understand the ancient remains and human activities.

## Data Availability

The datasets presented in this study can be found in online repositories. The names of the repository/repositories and accession number(s) can be found in https://www.ncbi.nlm.nih.gov/sra, PRJNA1188358.

## Footnotes

^1^ https://www.arb-silva.de/

^2^ https://unite.ut.ee/

^3^ https://cloud.majorbio.com
