# Supplementary material for: Insight into the microbial diversity and community in the sacrificial pits of Sanxingdui site (Sichuan, China)
Source: Front Microbiol. 2024 Dec 10;15:1489025. doi: 10.3389/fmicb.2024.1489025 (PMC11666563; doi:10.3389/fmicb.2024.1489025)
Supplement: Supplementary file 1 [file Supplementary_file_1.docx]

Supplementary Material

# Supplementary Figures and Tables

## Supplementary Figures


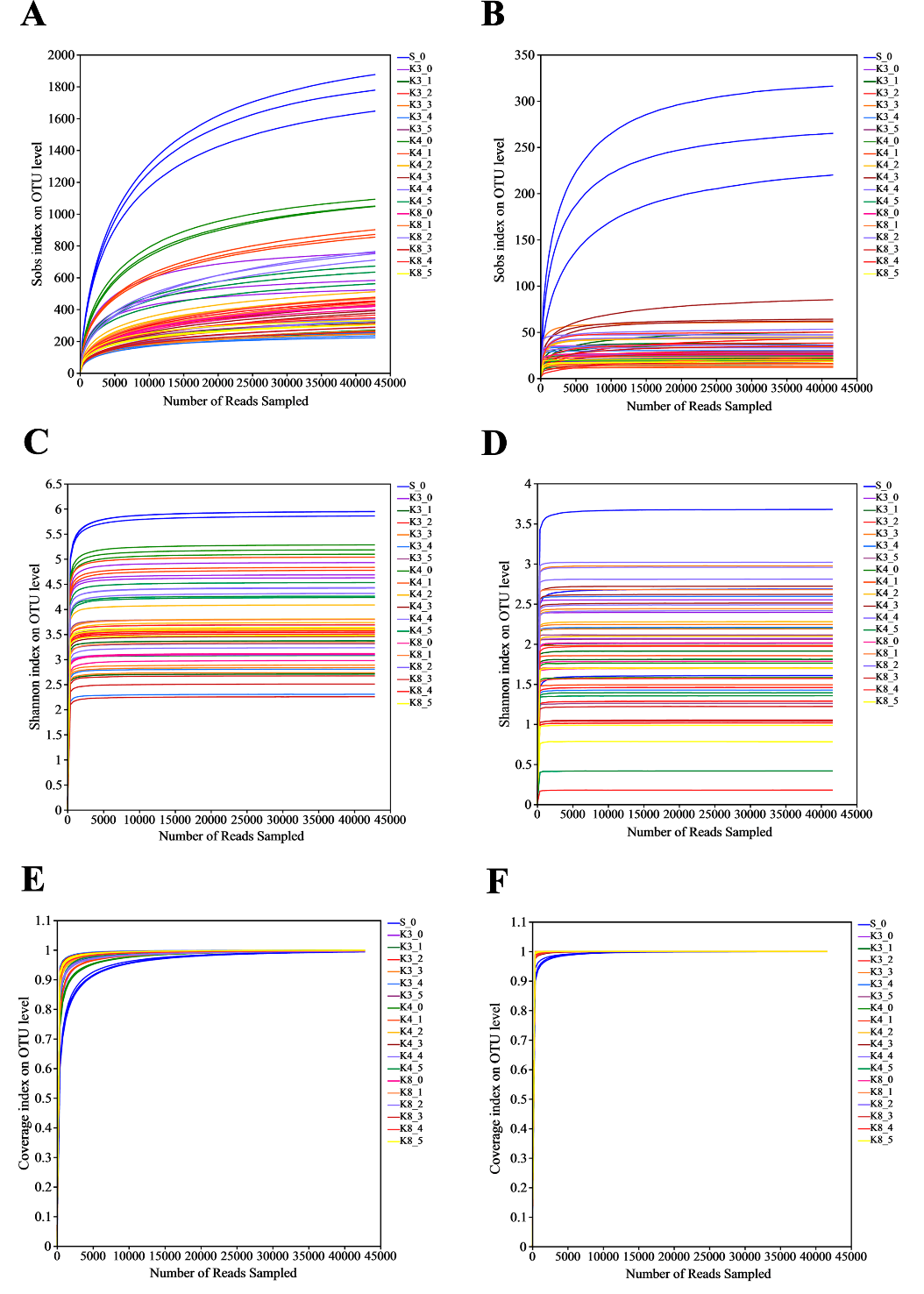


Figure S1 The rarefaction curves of soil samples. (A, B, C) Bacteria. (D, E, F) Fungi.


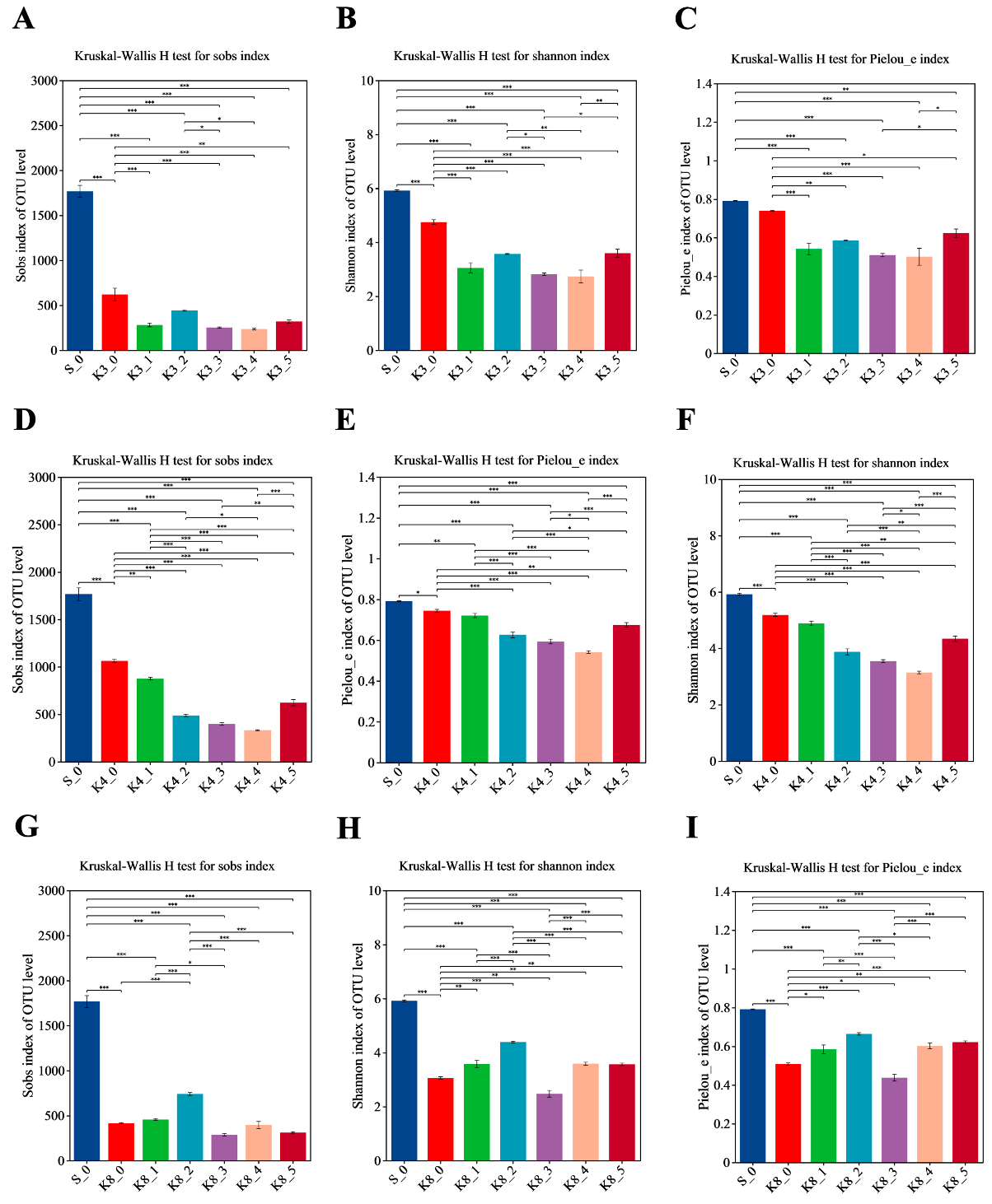


Supplementary Figure S2 The α-diversity of bacteria shown by Sobs, Shannon and Pielou_e. (A, B, C) Sacrificial pit K3. (D, E, F) Sacrificial pit K4. (G, H, I) Sacrificial pit K8.


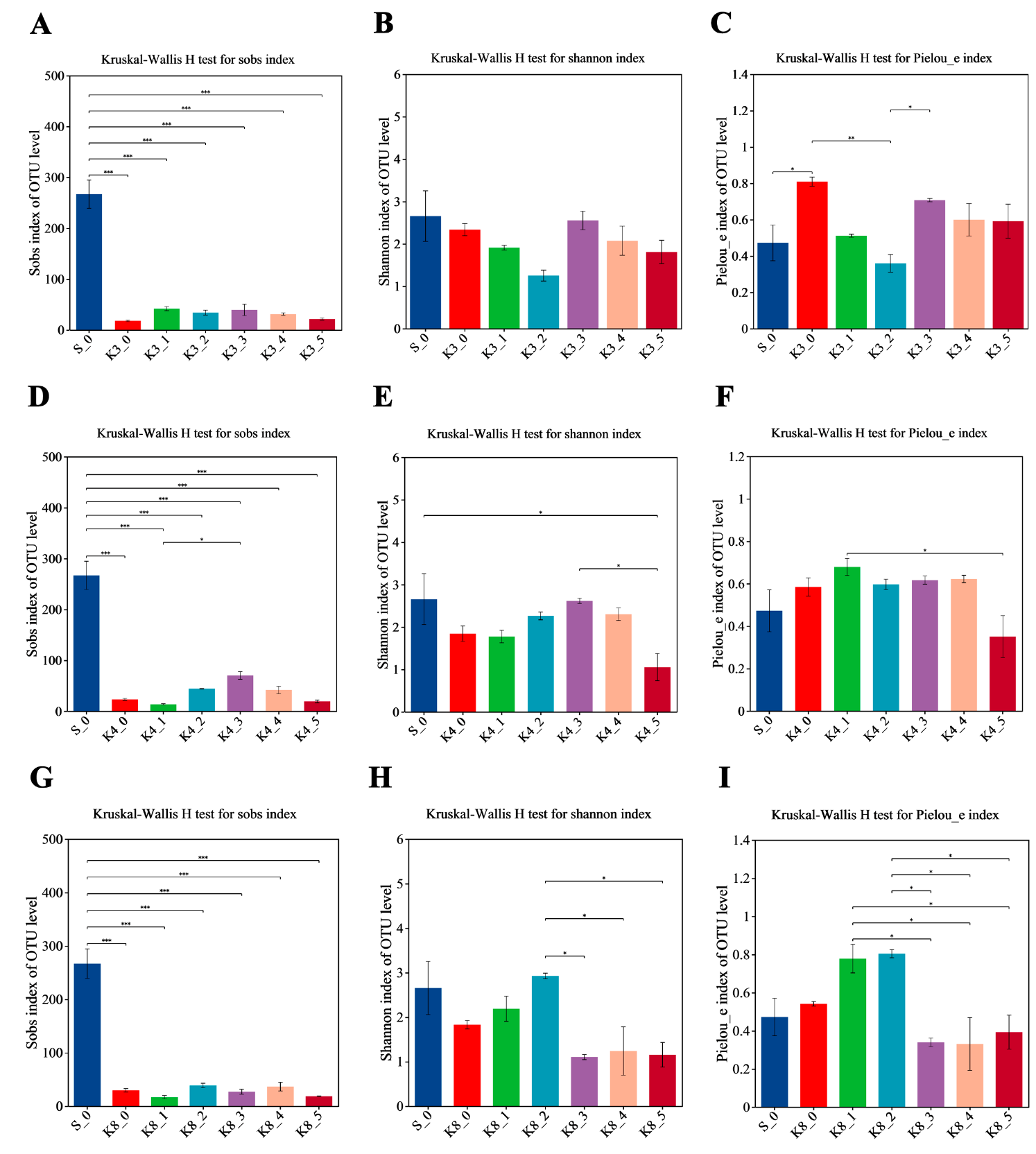


Supplementary Figure S3 The α-diversity of fungi shown by Sobs, Shannon and Pielou_e. (A, B, C) Sacrificial pit K3. (D, E, F) Sacrificial pit K4. (G, H, I) Sacrificial pit K8.


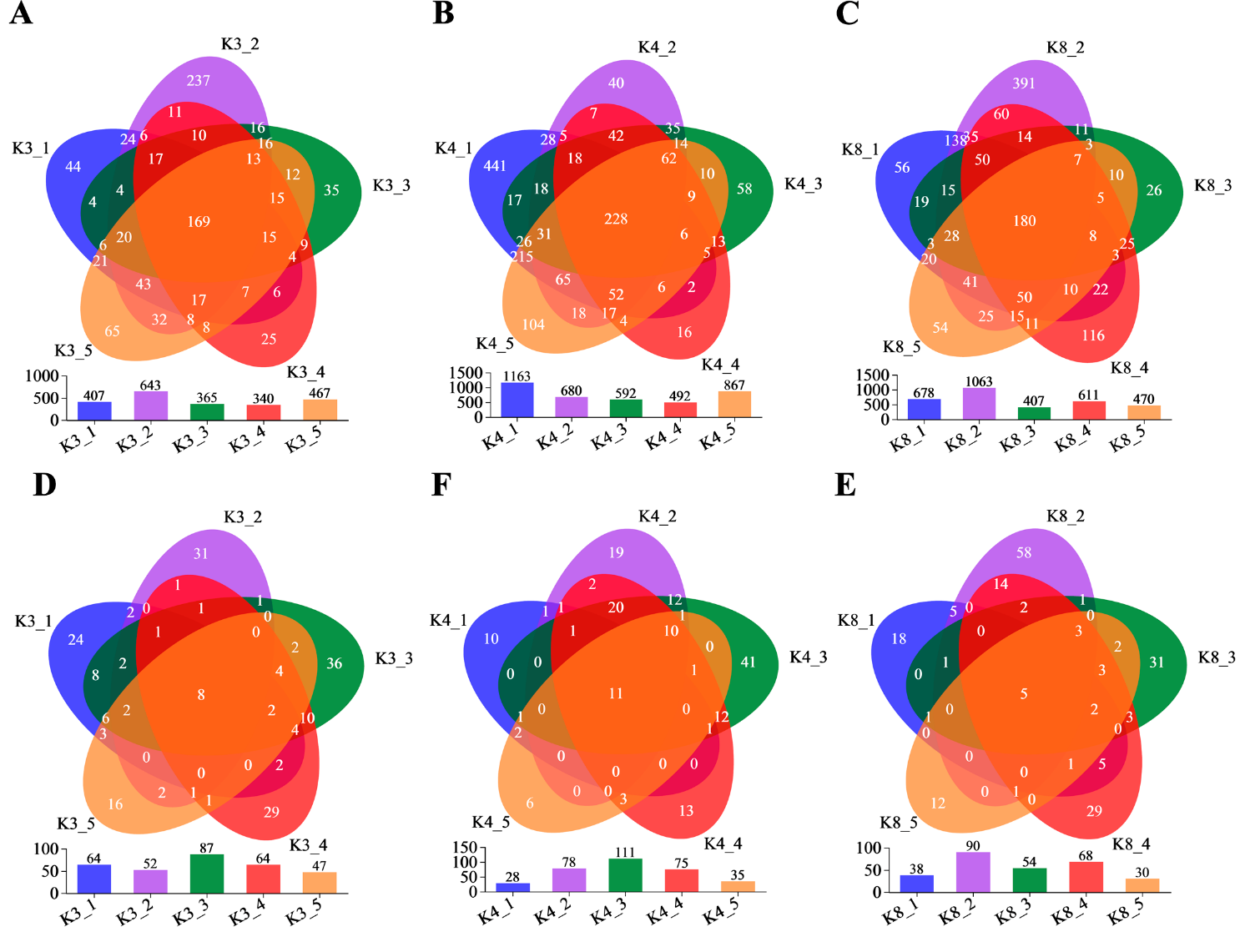


Supplementary Figure S4 Venn diagram on OTU level. (A, B, C) Bacteria. (D, E, F) Fungi.


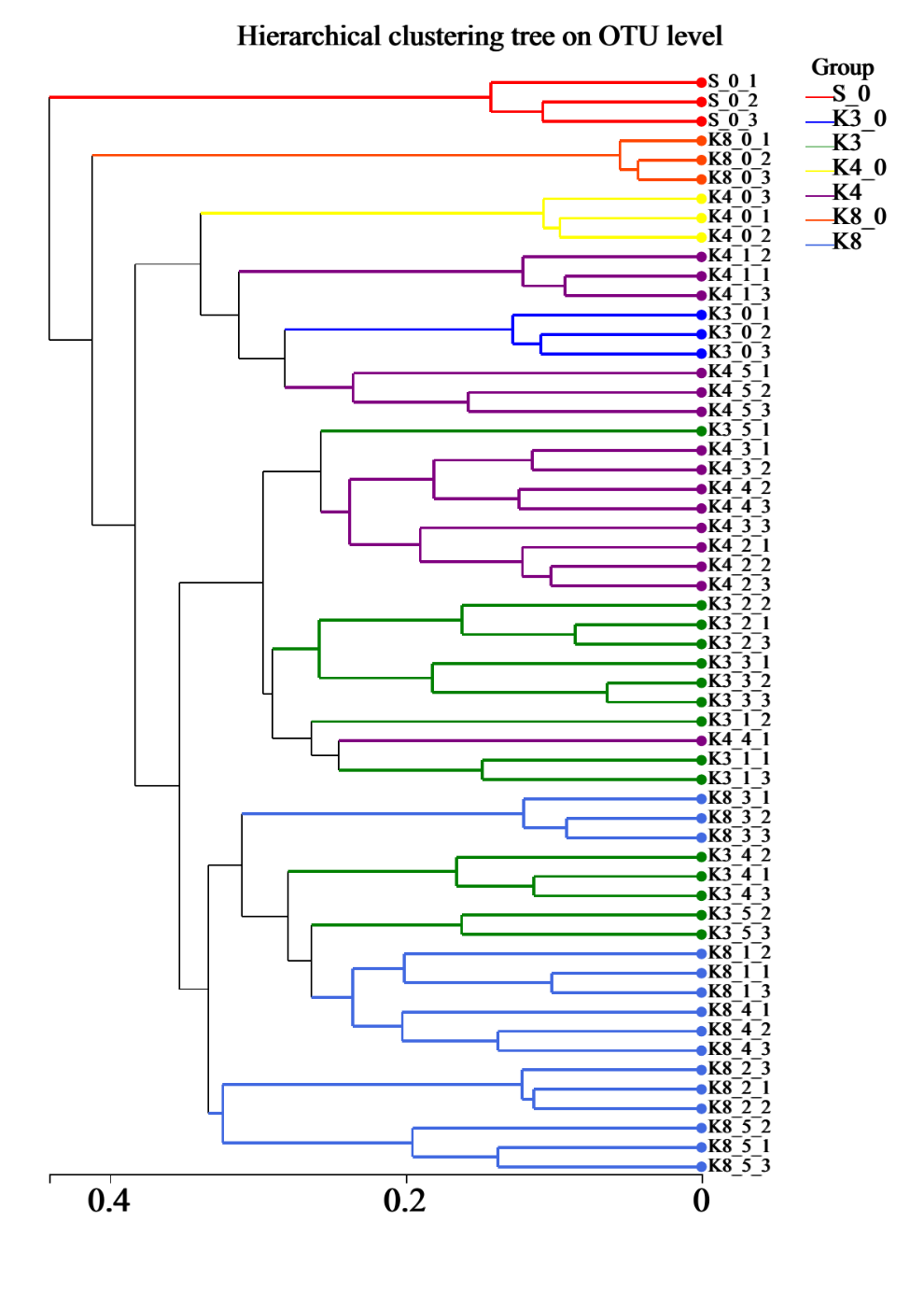


Supplementary Figure S5 The hierarchical clustering analysis (HCA) of bacteria in soil samples.


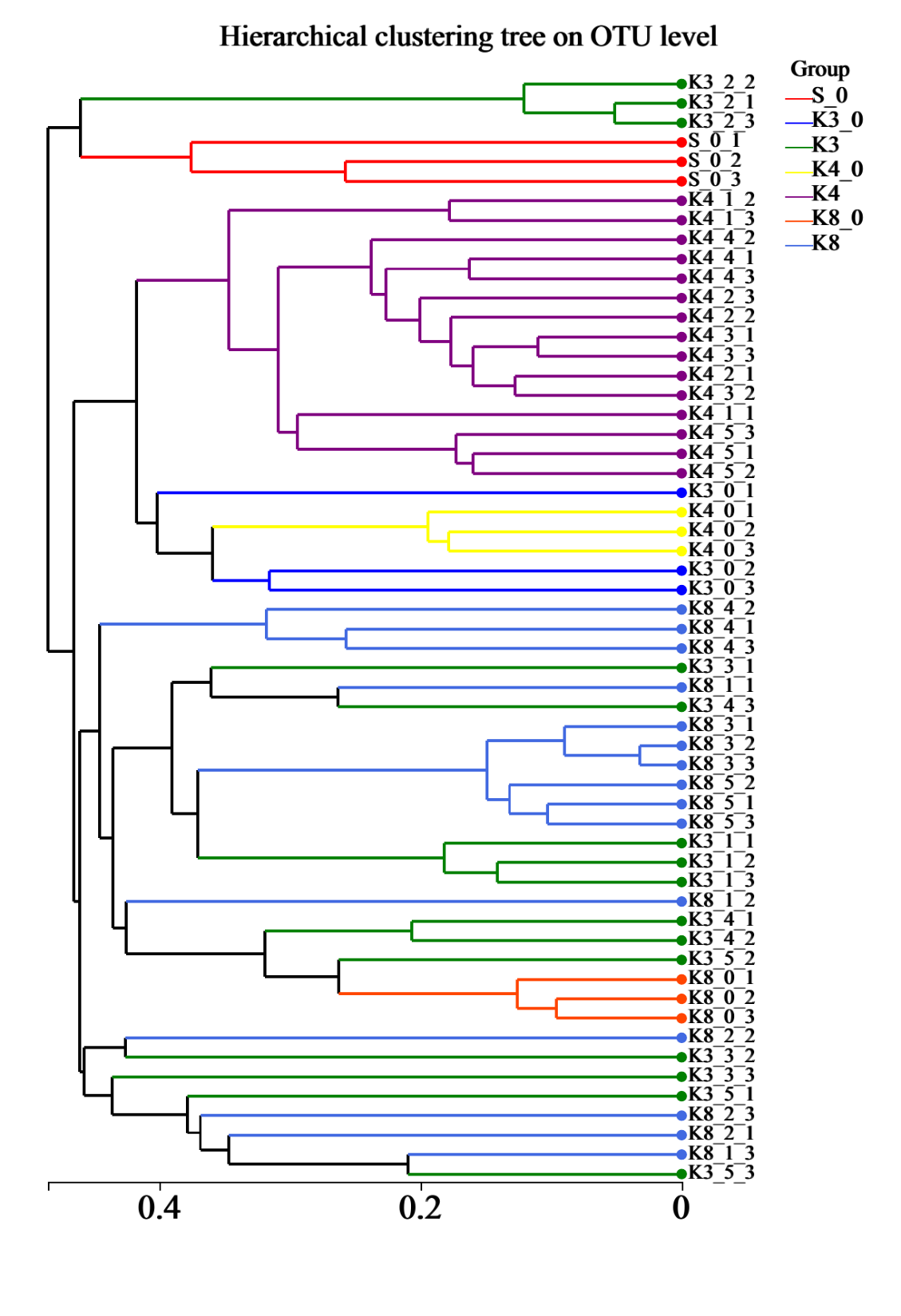


Supplementary Figure S6 The hierarchical clustering analysis (HCA) of fungi in soil samples.
